# The Combined Impact of Hand-Arm Vibration and Noise Exposure on Hearing Sensitivity of Agricultural/Forestry Workers—A Systematic Literature Review

**DOI:** 10.3390/ijerph20054276

**Published:** 2023-02-28

**Authors:** Ravinder Thaper, Richard Sesek, Richard Garnett, Yadrianna Acosta-Sojo, Gregory T. Purdy

**Affiliations:** Department of Industrial and Systems Engineering, Samuel Ginn College of Engineering, Auburn University, Auburn, AL 36849, USA

**Keywords:** hand arm vibration, noise exposure, hearing loss, chainsaw, forestry workers, threshold shift, combined exposure, Raynaud, vibration white finger

## Abstract

Hand-arm vibration (HAV), which potentially causes vibration white finger (VWF), and occupational noise are serious issues in the agricultural and forestry industries. Generally, agricultural workers operate as single-family/small businesses and thus are exempted from Occupational Safety and Health Administration (OSHA) regulations/laws for noise and HAV otherwise applicable to other industries in general. The agricultural/forestry sectors are at increased risk as working hours are longer than a typical 8-h work shift putting them at greater risk of hearing loss. The study was conducted to assess the possible association between hearing sensitivity on combined exposure to noise and hand-arm vibration. A systematic literature review was conducted on exposure to noise and HAV in the agricultural/forestry sector and the resulting impacts on hearing. The peer-reviewed articles in English were searched with 14 search words in three databases of PubMed, Ergo Abstracts, and Web of Science without any filter for the year for fully available article text. The database literature search resulted in 72 articles. Forty-seven (47) articles met the search criteria based on the title. Abstracts were then reviewed for any relationship between hearing loss and hand-arm vibration/Raynaud’s phenomenon/VWF. This left 18 articles. It was found that most agricultural workers and chainsaw workers are exposed to noise and VWF. Hearing is impacted by both noise and aging. The workers exposed to HAV and noise had greater hearing loss than non-exposed workers, possibly due to the additive effect on temporary threshold shift (TTS). It was found that VWF might be associated with vasospasm in the cochlea through autonomous vascular reflexes, digital arteries narrowing, vasoconstriction in the inner ear by noise, ischemic damage to the hair cells and increased oxygen demand, which significantly affects the correlation between VWF and hearing loss.

## 1. Introduction

Noise-induced hearing loss is a serious problem among United States workers affecting nearly 22 million people every year [1]. Virtually everyone is exposed to noise to some degree [2]. Health effects related to noise can be direct (auditory effects) and indirect (non-auditory) depending on the duration of exposure to sound signals and their intensity. Besides direct health effects leading to permanent effects such as hearing loss and tinnitus (ringing, buzzing etc., in the ears). The indirect health effects, such as sleep disorders with awakenings [3], learning impairment [4], hypertension, ischemic heart disease [5], diastolic blood pressure [6], reduction of working performance [7,8] and annoyance [9,10] are extremely important to consider. The scientific community is moving toward the prevention of these health effects [11].

Farmers and forestry workers are prone to hearing loss due to exposure to high noise levels for long work schedules because of seasonal job demands in the agricultural and forestry sectors. Approximately 37% of the workers suffer from hazardous noise levels, as illustrated by Agriculture, Forestry, Fishing, and Hunting Statistics (AFFH) [12]. Although farmers and forestry workers acknowledge they are exposed to hazardous noise levels, 27% reported not wearing hearing protection [12]. Audiograms of AFFH workers analyzed in the Surveillance Project study showed a significant decrease in hearing loss prevalence from 1981 (33%) to 2005 (13%), followed by an increase of 14% from 2006 to 2010. Overall, there was a significant decrease in incidence (11% to 6%) from 1986–2010. Even after these decreases in prevalence and incidence, it was stated that AFFH had the third highest hearing loss prevalence after Mining and the Healthcare and Social Assistance sectors [13].

Workers in the agricultural and forestry sectors are exposed to noise and vibration while using various kinds of handheld tools. The exposure to vibrations may cause Raynaud’s phenomenon, which occurs when there is a reduction of blood flow to fingers which results in a reduced tactile sense. The Vibration White Finger (VWF) is a secondary and severe form of Raynaud’s phenomenon, which results in cold blanching of fingers. Working frequently for long hours and exposure to vibrations simultaneously with noise increase the susceptibility to hearing loss [14,15,16]. The study conducted by Pykko et al., 1989 [17] discovered that forestry workers exposed to vibrations with noise had more severe hearing loss compared to other workers without vibration exposure. The objective of the study was to assess the combined synergistic effect of exposure to noise and hand-arm vibration on the hearing of forestry and agricultural sector workers.

## 2. Methods

### 2.1. Search Criteria

First, a systematic literature review protocol was developed. The search was carried out in three different databases (a) Web of Science, (b) PubMed, and (c) Ergo abstracts. Different combinations of words were chosen using ‘AND’ and ‘OR’ operators to identify articles relevant to the study. Finally, a string of words that resulted in the maximum number of relevant articles in searched databases for the purpose of the study was: ((Hand Arm Vibration OR Hand Vibration OR Arm Vibration OR Raynaud’s OR White Finger) AND Hearing AND (Agricultur* OR cultivat* OR lumberjack OR Chain Saw OR Farm* OR Forest* OR Sawyers OR Harvest*)).

### 2.2. Screening

An inclusion and exclusion strategy was defined. Inclusion criteria were: (a) peer-reviewed articles, (b) full text available, and (c) written in English. The exclusion criteria were: (a) articles undergoing the publishing process, (b) review papers, and (c) papers not written in English. The search was conducted without any filter for publication year. The items of interest in the title were exposure to hand-arm vibration, Raynaud’s phenomenon, VWF, and the resulting outcome of hearing loss in the agriculture sector, lumberjacks, chainsaw operators, and forestry workers. The articles that did not meet the above requirement in the title were not included in the study. After screening articles for titles, the abstracts were reviewed. The abstracts that did not present the relationship between hearing loss and hand arm vibration/Raynaud’s phenomenon/VWF were excluded. Finally, the articles meeting requirements were reviewed in full text and included in this study.

## 3. Results

**Figure 1:**
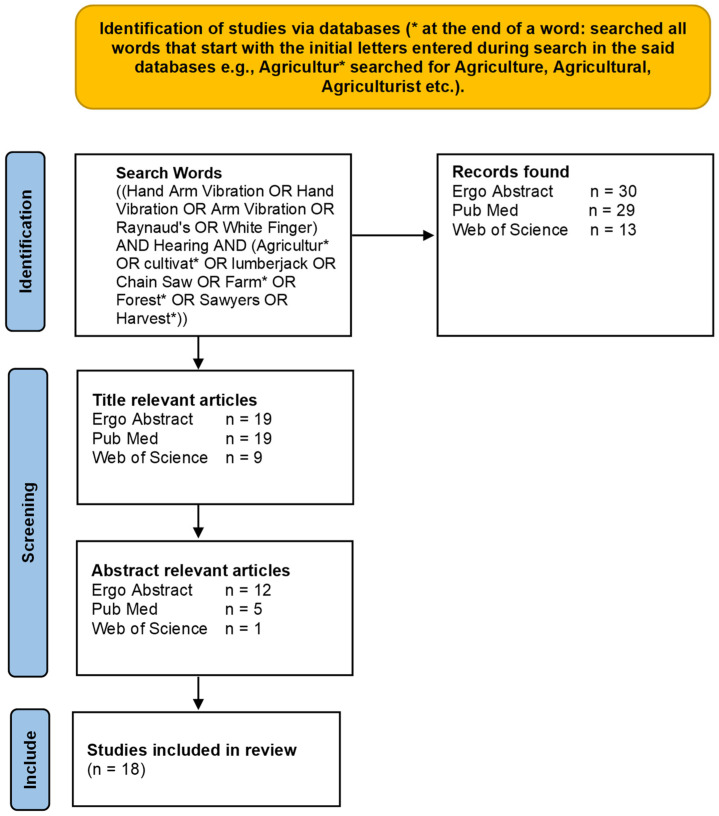
Flow diagram of article search results.

The literature search was conducted up to a publication date of November 2022 in databases of Ergo Abstracts, PubMed and Web of Science using the library’s subscription of the authors’ university. It resulted in 72 articles after removing duplicates and non-English language, as presented in Figure 1. Based on the initial search criterion of title relevance, 47 articles met the condition. Finally, article abstracts that demonstrated a relationship between hearing loss and hand-arm vibration/Raynaud’s phenomenon/vibration white finger were reviewed. This strategy reduced the number of articles to 18, which were thoroughly reviewed and included in this study.

**Figure 2:**
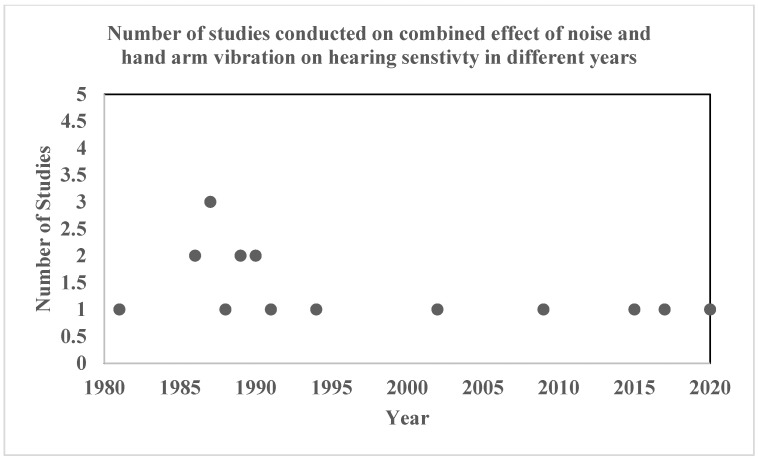
Number of studies conducted by year.

Figure 2 represents the number of studies published on the combined effect of noise and hand-arm vibration on hearing sensitivity at different points in time. The figure represents that 1987 had the highest number of studies conducted (three studies), followed by two studies in 1986, 1989, and 1990, and finally, one study in each of the other years. It can be said that very few researchers have studied the relationship/impact of the combined exposure of noise + hand-arm vibration on hearing sensitivity.

Table 1 shows most of the majority of studies (eight) were conducted in Japan, followed by Finland (five studies), and one study each in the remaining countries: USA, Italy, Canada, Lithuania, and Romania. It also shows the aim of each study, study type, number of subjects, subject age, inclusion and exclusion criteria, along with performance measures. Table 2 lists the standards used in each study, and Table 3 shows brief details of the findings.

## 4. Discussion

### 4.1. Association between Hand Arm Vibration (HAV) and Hearing Loss

Nine studies were found that established the relationship between HAV and hearing loss among forestry workers. The first study was conducted by Pyykko et al., 1981 [15]. The aim of the study was to determine the role of hand-arm vibration in the etiology of hearing loss in lumberjacks. A longitudinal study was conducted on Finnish forestry workers. Only the workers who had used chainsaws for a minimum of 500 hours per year were included in the study. It was found that hearing loss increased with age along with the duration of noise exposure and hearing protection use. The HAV-exposed subjects had significant hearing loss compared to non-HAV-exposed subjects with similar noise exposure.

The second study analyzed risk factors for sensory neural hearing loss during combined exposure to noise and vibration by Pyykko, Pekkarnine, and Starck in 1987 (Pyykkö, Pekkarinen, and Starck 1987). The VWF explained a 5% variation of SNHL in forestry workers at 4000 Hz. There was an interesting observation that the combination of noise and hand-arm vibration was not more hazardous to hearing than exposure to equivalent energy of noise alone. Iki et al., 1989 studied hearing of forestry workers affected by VWF with 5-year follow-up [23]. The noise-induced hearing loss was tested at 2, 4, and 8 kHz. The older subjects (>50 years) had greater hearing loss for tested frequencies compared to younger subjects (<50 years). The hearing loss was more severe in men with VWF regardless of age, hearing health, and noise exposure. The threshold shift at 8 kHz was similar in all age groups. However, hearing at 4 kHz was influenced by the interaction of noise exposure and VWF. Therefore, it was concluded that subjects exposed to noise and VWF were more vulnerable to hearing loss. The studies conducted by Iki et al. 1986; Iki 1994 and Pyykko et al., 1981 [15,18,27] discovered a similar association between VWF and hearing loss. 

Pyykko et al., 1989 [17] studied risk factors in the genesis of sensory neural hearing loss. They found that aging was the major factor for sensory neural hearing loss and explained 25% of the variance in sensory neural hearing loss, followed by noise exposure which explained 9% of the variance of sensory neural hearing loss.

Iki et al., 1990 [24] found that age was significant for every hearing level tested (500, 1000, 2000, 4000, and 8000 Hz). Chainsaw operation hours were significant for all hearing levels tested except for 8 kHz. The VWF had a significant correlation with hearing at 4 kHz. The hearing was found to be affected by both age and noise. Also, VWF was significantly correlated with hearing independent of age and noise exposure. The hearing of subjects without VWF, with VWF in both hands and VWF in one hand, were tested. No significant differences were observed in subjects with VWF in one hand and in both hands. At 4 kHz, in the subjects with VWF in one hand, significant differences were observed on the ipsilateral side of the hand with VWF, which was greater than the contralateral side. Laterality in hearing could be a problem in subjects with VWF in one hand, possibly due to the posture of the operator such that one ear was nearer to the noisy tool. The laterality of hearing loss might be due to different noise susceptibilities of both left and right ears on the ipsilateral and contralateral sides of the hand with VWF.

Murata, Araki and Aono, 1990 [25] discovered that chainsaw and brush saw operators both had moderate hearing loss for all frequencies tested. However, hearing was worse in chainsaw operators at 4 and 8 kHz. Turcot et al., 2015 [30] conducted research on noise-induced hearing loss and combined noise and vibration exposure. The researchers found that significant differences existed in hearing loss between forestry and mining workers with VWF and without VWF. There was a gradual decline in hearing in VWF subjects. The hearing was related to the duration of noise exposure. Working in environments with >90 dBA significantly contributed to the differential hearing deficit (DHD).

Iftime, Dumitrascu and Ciobanu, 2020 [32] conducted a study on chainsaw operators’ exposure to occupational risk factors and incidence of professional diseases specific to the forestry field. Exposure to high levels of noise, HAV, humidity and particulates might cause bilateral hearing loss and Raynaud’s syndrome (observed in 12% of workers with 26–35 years of forestry work experience). The diseases are closely related to age, work experience years in the current job, noise exposure, vibration, particulates and environment.

### 4.2. Possible Reasons for Hearing Loss Caused by Hand Arm Vibration (HAV)

The four studies conducted by Pyykko et al., 1981 [15], Pyykko et al., 1986 [19], Miyakita et al., 1987 [11] and Pyykko et al., 1989 [8] discussed the possible mechanism of hearing loss caused by combined exposure to noise and hand-arm vibration. Pyykko et al., 1981 [15] suggested vasoconstriction of the cochlear vessels triggered by the hand-arm vibration as one potential reason. If an individual with VWF is more susceptible to the ill effects of noise, then hearing might deteriorate further with digital vasospasms (vascular derangement with the sympathetic flow).

Another reason was suggested by Pyykko, Starck and Pekkarinen, 1986 [19] and Pyykko et al., 1989 [17]. The authors found that the simultaneous exposure to noise and vibration caused high energy demands. It caused the sympathetic nervous system to disturb the local compensatory changes in capillary beds by increasing circulation of the inner ear. This mechanism was responsible for hearing loss. This fact was supported by Miyakita et al., 1987 [20], who said that there was a relationship between hearing loss and peripheral circulation disorder due to the participation of the sympathetic nervous system found in some earlier animal studies. However, no relationship was established between hand-arm vibration exposure and cochlear microcirculation. Miyakita et al., 1987 stated that it was unclear if the reaction of microcirculation in the cochlea was analogous to digital vessels. The synergistic effect of noise and vibration might be considered a stressor that impacts temporary threshold shifts. The chainsaw operation demanded high energy. This energy demand activates the sympathetic nervous system, which might override the autoregulation of the inner ear and fingers as it disturbs the compensatory changes in the peripheral circulation.

### 4.3. Other Resulting Effects of Combined Exposure of Noise + Hand Arm Vibration

Chainsaw operation causes gradual decreases in finger skin temperature with cyclic changes corresponding to each exposure and break period, as found by researchers Pyykko, Starck and Pekkarinen, 1986 [19]. On the other hand, chainsaw handle temperature increased with increased operation times due to the running engine, as shown by Miyakita, Miura, and Futatsuka, 1987 [21]. The study conducted by Pyykko, Pekkarinen, and Starck, 1987 [14] on the analysis of risk factors for sensory neural hearing loss during combined noise and vibration exposure found that smoking did not appear to explain any significant variation in SNHL.

The VWF or skin temperature was found to be more significant in older persons (>50 years) in a study conducted by Iki et al., 1989 [23] on the hearing of forestry workers affected with VWF. It was found that cutaneous blood flow was regulated by the sympathetic nervous system, which was measured by skin temperature under controlled conditions. A slower skin temperature recovery after cessation of cold stimuli indicates that the nervous system reacted more intensely to cold stimuli. The researchers Pyyko et al., 1989 [17] studied risk factors in the genesis of SNHL. At 4000 Hz, a statistically significant correlation was found between VWF, serum LDL-cholesterol concentration, and use of antihypertensive drugs and SNHL which explained 28% of the variance in SNHL. The SNHL was observed to be not significantly correlated with salicylate medicine consumption, diastolic and systolic blood pressure, and smoking.

The combined effects of noise and HAV on auditory organ and peripheral circulation was studied by Miyakita, Miura, and Futatsuka, 1991 [26]. Three experimental conditions were used in the study: (1) subjects operated the chainsaw without any cutting involved, (2) subjects operated the chainsaw with double hearing protection (earmuffs and plugs), and (3) subjects stood beside someone operating the chainsaw. The researchers found that finger skin temperature decreased gradually during chainsaw operation. However, the handle temperature of the chainsaw increased. The finger skin temperature decreases considerably in Exposure 1 compared to Exposure 2 with more exposure time. The early stages did not show significant differences in skin temperature. No significant differences were observed for Exposure 1 and Exposure 3. The changes in blood flow had similar patterns to finger skin temperature. When the subjects operated the chainsaw at a higher working temperature, there was a significant reduction in finger skin temperature compared to the condition when subjects used double hearing protection.

The age and VWF exposure duration did not significantly correlate with average body sway velocity (ASV), as found by Iki, 1994 [27] while studying VWF as a risk factor for hearing loss and postural instability. Significant differences were found for ASV between the highest and lowest hearing subjects. The researchers Iftime, Dumitrascu and Ciobanu, 2020 [32] discovered that forestry workers revealed a high percentage of osteomusculoskeletal disorders (25.23%) when they conducted a study on forestry chainsaw operators’ exposure to occupational risk factors and incidence of professional diseases.

### 4.4. Noise and Hand Arm Vibration from Various Equipment

Chainsaws manufactured before 1970 caused hand-arm vibration between 10 and 20 ms^−2^ (root means square), and the later models after 1970 had 2 to 6 ms^−2^ [14]. The development of anti-vibrating chainsaws in 1972 produced a weighted acceleration of 1.8–2.2 ms^−2,^ along with features of reduced weight and impulsiveness of vibration. This made it possible for workers to increase tool usage exposure time to 5 h a day [22].

Neitzel and Yost [28] assessed different forestry tasks for noise and vibration in 2002. The average readings for National Institute Occupational Safety and Health (NIOSH) time-weighted average (TWA) sound level meter settings were 90.2 ± 5.1 dBA and 86.1 ± 6.2 dBA for Occupational Safety and Health-OSHA, respectively. The highest OSHA and NIOSH noises, TWA by operation, were observed in felling and road construction. Similarly, tree fellers and hook tenders are identified by job titles. The maximum whole-body vibration resulted from log processing (9.17 ms^−2^) and front-end loader (2.53 ms^−2^), respectively. The highest hand-arm vibration of 10.36 ms^−2^ was observed for tree felling. In the *x*-axis, the notching stump activity resulted in the highest HAV (8.12 ms^−2^). In the *y*-axis, the tree felling task resulted in the greatest HAV (5.64 ms^−2^). For the *z*-axis, chain idling produced the most HAV (6.95 ms^−2^). The chainsaw had the highest HAV A_eq_ with 6 ms^−2^, 4.26 ms^−2^ and 5.46 ms^−2^ for the x, y and z axes, respectively.

Monarca et al., 2009 [29] studied noise, vibration and dust in nut farms. The researchers found that the noise produced was >90 dB for various equipment such as towed harvesters, self-propelled harvesters, power-takeoff propelled harvesting devices (both hydraulic and mechanical), tractors, swathers, and blowers. Whole body vibration and hand-arm vibration for 8 h of exposure ranged from 0.21–0.97 ms^−2^ and 1.19–4.41 ms^−2^, respectively. Butkis and Vasiliauskas [31] evaluated farmers’ exposure to noise and vibration in small and medium-sized farms in 2017. The researchers reported that mean noise generated by combine harvesters, tractors with cabs, tractors without cab handheld tools for machine maintenance, handheld and guided tools for environmental works (lawnmowers, brush cutters, chainsaws etc.), grain equipment (augers, dryers, bin fans) and dairy farm equipment were 85.8, 87.3, 92, 94.3, 89.9, 86.2 and 82.4 dBA, respectively. Grain farming was observed to be louder than dairy farming. The noisiest activity in dairy farming was mechanized milking (87.1 dBA). For self-propelled, hand-guided and handheld machinery, the majority of HAV was in the range of 2.5–5 ms^−2,^ and 35% of the cases had 1.15 ms^−2^ for WBV. Air impact wrenches had the highest HAV of 8.9 ms^−2^, chainsaws and grinders > 5 ms^−2^ and impact hammers > 8 ms^−2^. For WBV, during cultivation unsuspended tractors generated the highest value of 2.86 ms^−2^ and combine harvesters with 2.19 ms^−2^.

### 4.5. Additional Observations

The use of earmuffs resulted in equivalent noise levels below 85 dB when used with helmet liners [22]. The helmet liners used by forestry workers increased the attenuation by 2–6 dBA. Earmuffs were found to attenuate high frequencies better than lower ones. Iftime, Dumitrascu and Ciobanu, 2020 [32] studied chainsaw operators’ exposure to occupational risk factors and incidence of professional diseases specific to the forestry field. The HAVs measured were associated with the wood type (soft or hard), the diameter of the tree, the topography of the land, chainsaw handling technique, wear and tear of equipment, capacity and dimensions of chainsaws, worker positioning while felling, cutting and trimming along with climatic conditions. The researchers Murata, Araki, and Aono, 1990 [25] said that the peak latency of brainstem auditory evoked potential-BAEP in chainsaw operators up to V-component, and interpeak latency between I and V was observed to be significantly longer. For brush saw operators, the peak latency (between I and V components) was significantly correlated with the number of years worked. No significant prolongation of BAEP latencies was found. The median nerve conduction for both chainsaw and brush saw operators was significantly slowed. The maximum voluntary contraction of brush saw operators was significantly correlated with years worked.

## 5. Conclusions

Hearing is impacted by noise and aging. Workers with VWF had greater hearing loss compared to workers without VWF. The combined exposure to noise and VWF made subjects more prone to hearing loss. The synergistic effect of noise and vibration appears to create a greater temporary threshold shift. The proposed mechanisms behind increased hearing loss in subjects exposed to simultaneous noise and VWF exposure are: (a) decreased blood flow resulting in ischemic damage and vibration damage transmitted via bone conduction due to prolonged and strong excitation of receptor cells, (b) digital arteries narrowing which results in increased oxygen demand, (c) hyper-responsiveness of the arteries to noradrenaline (d) changes in peripheral circulation and (e) hypersensitivity to catecholamine in a local median muscular layer along with hormonal effects. The sympathetic nervous system plays a major role in the genesis of VWF. VWF also explained some variance in postural stability and osteomusculoskeletal disorders. Exposure to hand-arm vibration could cause a significant decrease in finger skin temperature as well as musculoskeletal disorders. Modern chainsaws are quieter than older models. However, exposure times with chainsaws have increased as the vibration levels of newer chainsaws were reduced, making them more comfortable to use. Engineering controls such as acoustic treatment, enclosing engines, isolating heavy equipment operator compartments, and installation of more effective mufflers can reduce both noise and HAV. Despite the progress made in improving equipment and working conditions, noise remains a problem, particularly in agricultural and forestry settings.

## Figures and Tables

**Table 1:** Brief details of studies conducted by different researchers (NIPTS—Noise Induced Permanent Threshold Shift, SNHL—Sensory neural Hearing Loss, VWF—Vibration White Finger, NM—Not Mentioned, h-hours, WBV—Whole Body Vibration, NIHL—Noise Induced Hearing Loss, HAV—Hand Arm Vibration, ms^−2^—meter per second square, Leq—equivalent continuous sound level and dBA—decibels A scale adjusted to human hearing).

Reference	Origin	Title	Study Type	Subjects	Age (Years)	Inclusion Exclusion	Measures of Performance
Pyykko et al., 1981 [15]	Finland	Hand-arm vibration in the etiology of hearing loss in lumberjacks	Longitudinal	Lumberjacks (72 in 1972 to 203 in 1978)	20–50	Inclusion 1. Lumberjacks who used a chainsaw for at least 3 consecutive years with a minimum of 500 h a year Exclusion 1. NM	Exposure 1. Noise (Leq values 96–103 dBA) 2. HAV (linear acceleration 30–70 ms^−2^) Outcome 1. NIPTS
Iki et al., 1986 [18]	Japan	Association between vibration-induced white finger and hearing loss in forestry workers	Cross-sectional	Forestry workers (524 males and 19 females)	30–60	Inclusion 1. No history of ear disease, vertigo, head injury or drug intake that might impact hearing 2. Subjects not exposed to noise for 18 h before audiometric test Exclusion 1. Subjects < 30 years and >60 years	Exposure 1. Noise 2. HAV Outcome 1. Hearing loss 2. Recovery temperature of the skin in 5 minutes after cold provocation at 100 °C for 10 min
Pyykko, Starck and Pekkarinen, 1986 [19]	Finland	Further evidence of a relation between noise-induced permanent threshold shift and vibration-induced digital vasospasms	Longitudinal survey	32 forestry workers with digital vasospasms	20–50	Inclusion 1. Professional forestry workers who used a chainsaw for a minimum of 1500 h during the previous three years 2. A referent without VWF was symptomless forestry workers Exclusion 1. Forestry workers with ear disease or having noisy jobs other than sawing	Exposure 1. Noise 2. HAV Outcome 1. NIPTS
Miyakita, Miura and Futatsuka, 1987 [20]	Japan	Noise-induced hearing loss in relation to vibration-induced white finger in chainsaw workers	NM	499 operators of handheld vibration tools	40–69	Inclusion 1. No history of occupational noise exposure other than that of chainsaws 2. No history of ear disease, hearing abnormality, head injury, or the administration of drugs liable to affect hearing Exclusion 1. Subjects exposed to tool noise for at least 48 h prior to the audiometric test	Exposure 1. Noise 2. Vibration from chain saw Outcome 1. Association between NIHL and VWF
Pyykko, Pekkarinen and Starck, 1987 [14]	Finland	Sensory-neural hearing loss during combined noise and vibration exposure. An analysis of risk factors	NM	122 forestry workers	30–55	Inclusion 1. Noise and vibration exposure ranged from 6700 to 30,400 h Exclusion 1. Subjects with less than 1500 h of chain saw operating time in the last trimester	Exposure 1. Noise 2. HAV Outcome 1. SNHL
Miyakita, Miura and Futatsuka, 1987 [21]	Japan	An experimental study of the physiological effects of chainsaw operation	NM	19 healthy adult men (students, researchers, and clerks)	20–60	Inclusion 1. No previous exposure to the environmental factors Exclusion 1. NM	Exposure 1. Noise 2. HAV Outcome 1. Temporary threshold shift 2. Finger skin temperature
Starck, Pekkarinen and Pyykko, 1988 [22]	Finland	Impulse noise and hand-arm vibration in relation to sensory neural hearing loss	Longitudinal	199-forestry workers 171-shipyard workers	43.1 (Mean age for forestry workers 35.3 (Mean age for shipyard workers)	Inclusion 1. Forestry and shipyard workers Exclusion 1. Workers with hearing loss caused by disease or accident	Exposure 1. Noise 2. HAV Outcome 1. SNHL
Iki et al., 1989 [23]	Japan	Hearing of forest workers with vibration-induced white finger—a 5-year follow-up	Cross-sectional	86 male forestry workers	48.4 (mean age at the beginning of the study)	Inclusion 1. Male forestry workers who regularly use vibrating tools Exclusion 1. No history of noise exposure other than that from chainsaws, bush cleaners or winches 2. No history of ear diseases, vertigo, head injury, or the intake of ototoxic drugs 3. No conductive hearing loss	Exposure 1. Noise 2. HAV Outcome 1. Hearing loss
Pyyko et al., 1989 [17]	Finland	Risk factors in the genesis of sensorineural hearing loss in Finnish forestry workers	Longitudinal	199 forestry workers	25–60	Inclusion 1. Mean exposure to chainsaw noise for 15–370 h Exclusion 1. NM	Exposure 1. Noise 2. VWF Outcome 1. Hearing loss
Iki et al., 1990 [24]	Japan	Vibration-induced white finger and auditory susceptibility to noise exposure	Cross-sectional study	88 male workers	48.1 (mean age)	Inclusion 1. No history of ear diseases, vertigo, head injury, or the intake of ototoxic drugs 2. No continuous use of hearing protectors 3. No noisy hobbies other than chainsaws, bush cleaners or winches 4. No conductive hearing loss was detected by otoscopy and audiometry Exclusion Subjects with age < 30 years and >60 years	Exposure 1. Noise 2. VWF Outcome 2. Hearing loss
Murata, Araki, and Aono, 1990 [25]	Japan	Central and peripheral nervous system effects of hand-arm vibrating tool operation A study of brainstem auditory-evoked potential and peripheral nerve conduction	NM	12 chainsaw and 8 brush saw operators Controls-52 healthy male adults without otitis, deafness and tinnitus, living in the same residential area as the vibration operators were not engaged either in occupations using vibrating tools or suffering from endocrinological or neurological disorders	44–63 (chainsaw operators) 23–56 (Brush saw operators)	Inclusion 1. Male chainsaw and bush operators Exclusion NM	Exposure 1. Noise 2. VWF Outcome 1. Hearing loss
Miyakita, Miura and Futatsuka, 1991 [26]	Japan	Combined Effects of Noise and Hand-Arm Vibration on Auditory Organ and Peripheral Circulation	NM	Study 1: 19 healthy adult males Study 2: 12 healthy adult males	Study 1: 20–60 Study 2: 20–50	Inclusion 1. Adult healthy males Exclusion 2. No previous exposure to environmental factors involved	Exposure 1. Noise 2. VWF Outcome 1. Temporary threshold shift 2. Change in finger skin temperature
Iki 1994 [27]	Japan	Vibration-induced white finger as a risk factor for hearing loss and postural instability	Longitudinal	Study 1: 289 Japanese forestry workers Study 2: 71 Finnish forestry workers	Study 1: 48 Study 2: 42.9	Study 1: Inclusion Forestry workers Exclusion History of ear diseases, exposure to ototoxic factors except for noise Study 2: Inclusion Forestry workers Exclusion Diseases that might affect postural stability	Study 1: Exposure 1. Noise 2. VWF Outcome 1. Hearing loss Study 2: Exposure 1. VWF Outcome 1. Postural stability
Neitzel and Yost, 2002 [28]	USA	Task-based assessment of occupational vibration and noise exposures in forestry workers	NM	43 forestry workers	47	Inclusion Forestry workers Exclusion NM	Exposure 1. Noise 2. WBV 3. HAV Outcome 1. Noise 2. WBV 3. HAV
Monarca et al., 2009 [29]	Italy	Safety and Health of Workers: Exposure to Dust, Noise and Vibrations	NM	NM	NM	Inclusion 1. Nut farm workers Exclusion 1. NM	Exposure 1. Noise 2. Vibration Outcome 1. Noise 2. HAV and WBV
Turcot et al., 2015 [30]	Canada	Noise-induced hearing loss and combined noise and vibration exposure	Cohort	15,757 vibration-exposed workers (96 HAV exposed)	25–64	Inclusion 1. Forestry and mining workers exposed to >80 dBA Exclusion 1. NM	Exposure 1. Noise 2. Vibration Outcome 1. Hearing loss 2. HAV
Butkus and Vasiliauska, 2017 [31]	Lithuania	Farmers’exposure to noise and vibration in small and medium-sized farms	NM	Workers in 14 small and medium-sized farms	NM	Inclusion 1. Medium and small-sized farms involved in tractor driving, cultivation activities, transportation, animal feed preparation works, grain harvesting, grass cutting with brush cutters, lawnmowers as well as using chainsaws, circular saws, angle grinders, impact drills, air impact wrenches etc. Exclusion 1. NM	Exposure 1. Noise 2. Vibration Outcome 1. Noise 2. HAV and WBV
Iftime, Dumitrascu and Ciobanu, 2020 [32]	Romania	Chainsaw operators’ exposure to occupational risk factors and incidence of professional diseases specific to the forestry field	NM	107 chainsaw operators	21–62	Inclusion 1. Chainsaw operators Exclusion 1. Occupational disease	Exposure 1. Noise 2. Vibration 3. Dust Outcome 1. Noise 2. HAV 3. Wet bulb globe temperature 4. Dust

**Table 2:** Standards followed in each study (NM—Not Mentioned, WBV—Whole Body Vibration, HAV—Hand Arm Vibration).

Reference	Research Objective	Standards Used
Pyykko et al., 1981 [15]	Hand-arm vibration in the etiology of hearing loss in lumberjacks	1. American National Standard Institute (ANSI). The relation of hearing loss to noise exposure. New York: ANSI, 1954:64. Report by exploratory subcommittee-Z24-X-2. 2. International Electrotechnical Commission. Precision sound and workers. Geneva: IEC, 1973. Publication 179.3. International Electrotechnical Commission. Octave, half-octave and third-octave band filters intended for the analysis of sounds and vibrations. Geneva: IEC, 1966. Recommended publication 225. 4. Economic Commission for Europe/Food and Agricultural Organization. International Labour Organization, the joint committee on forest workers, technique, and training. Hand-operated chainsaws with internal combustion engine protection against vibration diseases. Part II: Operator-held measurement method. Geneva: ILO, 1975.5. International Organization for Standardization (ISO). Reference zero for the calibration of pure-tone audiometers. Geneva: ISO, 1964. ISO recommendation 389. 6. International Organization for Standardization (ISO). Draft international standard ISO/DIS 5349. Principles for the measurement and the evaluation of human exposure to vibration transmitted to the hand. Geneva: ISO, 1978.
Iki et al., 1986 [18]	Association between vibration-induced white finger and hearing loss in forestry workers	1. Japanese Industrial Standard. Diagnostic audiometers. Tokyo 1963. (JIS T 1201-1963). 2. International Organization for Standardization. Acoustics-Standard reference zero for calibration of pure tone audiometers. Geneva 1975. (ISO 389).
Pyykko, Starck and Pekkarinen, 1986 [19]	Further evidence of a relation between noise-induced permanent threshold shift and vibration-induced digital vasospasms	1. ISO DIS 5349. Principles for the measurement and the evaluation of human exposure to vibration transmitted to the hand, Geneva, International Organization for Standardization, 1978. 2. ISO DIS 5349. Guide for the measurement and assessment of human exposure to vibration transmitted to the hand. Geneva, International Organization for Standardization, 1984. 3. IEC 651. Precision Sound Level Meters Publication 651. Geneva, International Electrotechnical Commission, 1979. 4. IEC 225. Octave, half-octave and third-octave band filters for the analysis of sound and vibrations, Geneva, International Electrotechnical Commission, 1966. 5. ISO/R 1996. Acoustics—assessment of noise with respect to community response, Geneva, International Organization for Standardization, 1971. 6. ISO 389. Acoustics standard reference zero for the calibration of pure-tone audiometers. Geneva, International Organization for Standardization, 1975.
Miyakita, Miura and Futatsuka, 1987 [20]	Noise-induced hearing-loss in relation to vibration-induced white finger in chainsaw workers	1. Audiometer calibration- Japanese Industrial Standards. Diagnostic audiometers. Tokyo 1963. (JI S T 1201–1963). 2. American National Standards Institute. ANSI criteria for permissible ambient noise during audiometric testing. Section 3.1. New York, NY 1977.
Pyykko, Pekkarinen and Starck, 1987 [14]	Sensory-neural hearing loss during combined noise and vibration exposure. An analysis of risk factors	1. Vibration acceleration—ISO 5349 (1986) International standard Guidelines for the measurement and the assessment of human exposure to hand-transmitted vibration International Organization for Standardization, p. 13. 2. Noise—ISO/DIS 1999 2 (1985) Draft international standard Acoustics-determination of occupational noise exposure and estimation of noise-induced hearing impairment International Organization for Standardization, p. 9.
Miyakita, Miura and Futatsuka, 1987 [21]	An experimental study of the physiological effects of chainsaw operation	Sound level meter (Rion NA 60) with octave filters the demands of the IEC standard.
Starck, Pekkarinen and Pyykko, 1988 [22]	Impulse noise and hand-arm vibration in relation to sensory neural hearing loss	1. Finnish Standards Association. Kuulonsuojaimet: Vaatimukset [Hearing protectors: Requirements]. Helsinki 1979. (SFS 4431). 2. Finnish Standards Association. Kuulonsuojaimet:Testaus [Hearing protectors: Test methods]. Helsinki 1979. (SFS 4432). 3. International Organization for Standardization. Acoustics—Measurement of sound attenuation of hearing protectors—Subjective method. Geneva 1981. (International standard ISO 4869). 4. International Organization for Standardization. Acoustics—Determination of occupational noise exposure and estimation of noise-induced hearing impairment. Geneva 1985. (ISO/DIS 1999.2). 5. International Organization for Standardization. Guidelines for the measurement and the assessment of human exposure to hand-transmitted vibration. Geneva 1986. (International standard ISO 5349).
Iki et al., 1989 [23]	Hearing of forest workers with vibration-induced white finger-a 5-year follow-up	Audiometer calibration-Japanese Industrial Standard (JIS T1201-1982), which has the same zero levels as the international standard (ISO 389-1975)
Pyyko et al., 1989 [17]	Risk factors in the genesis of sensorineural hearing loss in Finnish forestry workers	NM
Iki et al., 1990 [24]	Vibration-induced white finger and auditory susceptibility to noise exposure	Audiometer calibration- Japanese Industrial Standard (JIS T 1201-1982), which has the same zero levels as the international standard (ISO 389-1975)
Murata, Araki, and Aono, 1990 [25]	Central and peripheral nervous system effects of hand-arm vibrating tool operation. A study of brainstem auditory-evoked potential and peripheral nerve conduction	NM
Miyakita, Miura and Futatsuka, 1991 [26]	Combined Effects of Noise and Hand-Arm Vibration on Auditory Organ and Peripheral Circulation	NM
Iki 1994 [27]	Vibration-induced white finger as a risk factor for hearing loss and postural instability	NM
Neitzel and Yost, 2002 [28]	Task-based assessment of occupational vibration and noise exposures in forestry workers	1. WBV in 3 mutually perpendicular axes (x, y, and z)—International Organization for Standardization (ISO) standard 2631/1–1985, using a 1-s time constant. 2. HAV Triaxial basicentric rms acceleration- ISO 5349–1986, using a 1-s time constant. 3. Commission of the European Communities (CEC): COM(92) 560—Final: Proposal for a Council Directive on the Minimum Health and Safety Requirements Regarding the Exposure of Workers to the Risks Arising from Physical Agents. Brussels, Belgium: CEC, 1992.
Monarca et al., 2009 [29]	Safety and Health of Workers: Exposure to Dust, Noise and Vibrations	1. Noise-Decree law 195/06, Italian reception of the directive 2003/10/ CE. 2. WBV and HAV-Decree law 187/05, Italian reception of the directive 2002/44/CE, based on norms ISO 5349 and ISO 2631.
Turcot et al., 2015 [30]	Noise-induced hearing loss and combined noise and vibration exposure	1. Hearing loss not attributable to age- International Organization for Standardization (ISO) 7029.
Butkus and Vasiliauska, 2017 [31]	Farmers’exposure to noise and vibration in small and medium-sized farms	1. Noise- International standards ISO 9612, ISO 5349-1, ISO 5349-2, ISO 2631-1 and ISO 2632-2. 2. EU directive for occupational noise (2003/10/EC) and vibration (2002/44/EC)
Iftime, Dumitrascu and Ciobanu, 2020 [32]	Chainsaw operators’ exposure to occupational risk factors and incidence of professional diseases specific to the forestry field.	1. Vibration- ISO 5349-1:2001 2. Government Decision (GD). Minimum safety and health requirements regarding the exposure of workers to the risks arising from noise. Bucharest: Romanian Official Gazette; 2006. GD no. 493/2006. Romanian. 3. Government Decision (GD). Modification and completion of normative acts in the field of health and safety at work. Bucharest: Romanian Official Gazette; 2007. GD no. 601/2007. Romanian. 4. Government Decision (GD). Minimum safety and health requirements regarding the exposure of workers to the risks from vibration. Bucharest: Romanian Official Gazette; 2005. GD no. 1876/2005. Romanian. 5. Government Decision (GD). Modification and completion of normative acts in the field of health and safety at work. Bucharest: Romanian Official Gazette; 2015. GD no. 359/2015. Romanian. 6. Law no. 319/2006. Health and safety at work act]. Bucharest: Romanian Official Gazette; 2006. GD no. 646/2006. Romanian. 7. Council Directive 89/391/EEC of 12 June 1989 on the introduction of measures to encourage improvements in the safety and health of workers at work. OJ. 1989; L183:1–8. 2008.

**Table 3:** Brief Summary of Study Findings (HAV—Hand Arm Vibration, WBV—Whole Body Vibration, NIPTS—Noise Induced Permanent Threshold shift, HL—Hearing Loss, TTS—Temporary Threshold Shift, VWF—Vibration White Finger, SNHL—Sensory Neural Hearing loss, ms^−2^—meter per second square, kHz—kilohertz, °C—degree Celsius, ASV—Average Body Sway Velocity, NM—Not Mentioned, DBP—Diastolic Blood Pressure, mgm^−3^—milligram per cubic meter, OSHA—Occupational Safety and Health Administration, NIOSH—National Institute for Occupational Safety and Health and TWA—Time Weighted Average).

Reference	Findings	Evidence Supporting the Objective of the Study
Pyykko et al., 1981 [15]	1. 1960s chainsaws had an average noise level of 111 dB(A) and acceleration of 60–180 ms^−2^. 2. VWF resulted in 10 dB greater NIPTS compared to subjects without VWF. 3. Chainsaw noise exposure increased NIPTS. For equal noise exposure, 10 dB greater NIPTS was observed in lumberjacks with VWF compared to those without VWF exposure. 4. Lumberjacks with VWF consistently had 10 dB greater NIPTS than those without VWF for the same duration of noise exposure.	NIPTS
Iki et al., 1986 [18]	1. Older subjects had higher hearing loss at 4 and 8 kHz. The greatest hearing loss was observed at 4 kHz, which is typical of noise-induced hearing loss. 2. Hearing was observed to be impacted by noise and aging. 3. Subjects exposed to VWF has significantly higher hearing threshold compared to controlled ones at 4 and 8 kHz. 4. The group of men with a mean age of 50.2 years had the highest recovery rate of finger skin temperature 5 min after cold provocation at 10 °C for 10 min compared to the group with 52.6 years of age, which observed the lowest recovery rate.	HL
Pyykko, Starck and Pekkarinen, 1986 [19]	1. The chainsaws manufactured after 1970 had reduced vibrations (tenfold) with a slight reduction in noise. 2. Digital vasospasms contributed to NIPTS.	NIPTS
Miyakita, Miura and Futatsuka, 1987 [20]	1. The peak values for chainsaw vibrations ranged from 8 to 14 ms^−2^. 2. The peak values of the sound pressure level ranged from 105 to 118 dBA. 3. Significant hearing loss was observed in the chainsaw workers with VWF compared to the ones without VWF. 4. No significant differences in hearing loss were observed within VWF-exposed and non-exposed for 10-to-l4-years exposure. 5. For 50-to 59-years age groups, significant differences were observed for hearing loss. 6. The study suggested that synergistic effects of noise and vibration might be due to the inter individual differences in the susceptibility to noise and vibration.	NIHL
Pyykko, Pekkarinen and Starck, 1987 [14]	1. Aging explained 15.4% of the variance of the SNHL (major risk factor). 2. VWF explained 5.2% of the SNHL (the second most important risk factor). 3. Elevation of DBP explained 4.1% of SNHL and correlated significantly with SNHL. 4. Smoking and systolic blood pressure did not significantly contribute to the genesis of SNHL. 5. No exaggerated risk of SNHL was observed for the combination of noise and vibration 6. Combined VWF and elevated DBP ran a higher risk for SNHL.	SNHL
Miyakita, Miura and Futatsuka, 1987 [21]	1. Exposure 1 (higher chainsaw speed) caused more reduction in mean normalized finger skin temperature with increased exposure time compared to when the chainsaw operated using exposure 2 (double hearing protection). 2. Exposure 1 caused a significantly greater temporary threshold shift than at 4 kHz compared to exposure 3 (when the subject stood beside someone else operating a chainsaw). 3. Noise and vibration exposure might cause constriction of the peripheral vessels. 4. Hand-arm vibration might produce an additive effect on the noise-induced temporary threshold shift.	TTS
Starck, Pekkarinen and Pyykko, 1988 [22]	1. Forestry and shipyard workers had nearly equal exposure to noise measured outside the earmuffs over time. 2. Shipyard workers had greater impulse noise outside and inside earmuffs compared to forestry workers. 3. The average SNHL was observed to be higher than predicted in shipyard workers and roughly about the same for forestry workers. 4. The total earmuff wearing time influenced the exposure level inside the earmuffs. 5. The earmuffs were not found to attenuate low frequencies of the chainsaw noise sufficiently. 6. The study found that exposure to impulse noise increased the risk of SNHL. 7. The combined exposure to hand-arm vibration and noise was not observed to increase the risk of SNHL.	SNHL
Iki et al., 1989 [23]	1. The forestry workers exposed to VWF were found to be more vulnerable to noise. 2. The pathological change responsible for VWF was also observed to possibly contribute to hearing loss.	HL
Pyyko et al., 1989 [17]	1. The major risk factors for hearing loss were found to be aging, followed by noise exposure and VWF. 2. The concentration of plasma low-density lipoprotein cholesterol-LDL and antihypertensive drug use were observed to be significantly correlated with SNHL. 3. The main factors (aging, noise exposure, VWF, LDL cholesterol and antihypertensive drug use) explained about 28% of the SNHL variance. 4. Other factors, such as smoking, systolic and diastolic blood pressure, and salicylate consumption, did not significantly influence SNHL.	HL
Iki et al., 1990 [24]	1. The exposure to VWF caused more hearing loss at 4 and 8 kHz compared to non-exposed subjects. 2. At 4 kHz, the subjects with VWF on the ipsilateral side of the hand had greater hearing loss than subjects having a contralateral side with VWF in one hand. 3. During the five years follow-up period, the hearing loss at 4 kHz progressed more rapidly in the subjects with VWF than in those with no history of VWF. 4. It was concluded that exposure to VWF made the subjects more prone to hearing loss. 5. Enhanced vasoconstriction due to elevated sympathetic nervous tone caused VWF. It was suggested that this could cause additional auditory vulnerability to noise exposure.	HL
Murata, Araki, and Aono, 1990 [25]	1. The combined effect of different stressors, local vibration (vibrating tool operation), noise, climate and heavy work affected not only the peripheral nervous system but also the brainstem portion of the auditory pathway. 2. Significant prolonging was observed for I-V interpeak latency (conduction from cochlear nerve to brainstem) and V peak latency of brainstem auditory-evoked potential-BAEP in chainsaw operators. 3. The number of working hours with brush saw operators were observed to be significantly associated with I-V interpeak latency.	HL
Miyakita, Miura, and Futatsuka, 1991 [26]	1. The chainsaw workers with VWF had significantly greater hearing loss at high frequencies compared to those without VWF. 2. It was suggested that noise and HAV might constrict the peripheral vessels, thus producing an additive effect on the noise-induced temporary threshold shift.	TTS
Iki 1994 [27]	1. At 4 kHz and 8 kHz, the subjects with VWF had a significantly higher hearing threshold compared to the controls. 2. At 4 kHz, a significant positive correlation was found between ASV and hearing. 3. The age, duration of noise exposure and chainsaw vibration didn’t correlate with ASV. 4. The workers with VWF were found to have greater hearing loss at 4 kHz and 8 kHz. The hearing loss significantly correlated with ASV.	HL
Neitzel and Yost, 2002 [28]	1. Substantial overexposure to noise was observed in forestry workers. The mean OSHA TWA was 86.1 ± 6.2 dBA. The mean NIOSH TWA is 90.2 ± 5.1 dBA. 2. The mean weighted HAV was observed to be 5.45 ± 5.25 ms^−2^. 3. The mean weighted whole-body vibration was 3.53 ± 7.12 ms^−2^. 4. The highest exposure activities and tools were unbelling chokers and chainsaws (noise), log processing and front end loader (WBV), and notching stumps and chainsaws (HAV).	-
Monarca et al., 2009 [29]	1. The noise exposure was >90 dBA which might affect mental functions, hearing, and human anatomical and physiological structures. 2. The vibrations produced by different blower models were found to be in the range of 0.21–0.97 ms^−2^ and 1.19–4.41 ms^−2^ for WBV and HAV, respectively.	-
Turcot et al., 2015 [30]	1. Greater hearing loss at higher frequencies was observed in workers with VWF. 2. An association exists between HAV and hearing loss.	HL
Butkus and Vasiliauska, 2017 [31]	1. The noise exposure level exceeded the action value of 80 dBA. 2. Hand-arm vibration and whole-body vibration exposure were observed to be 1.15 and 5 ms^−2^, respectively.	-
Iftime, Dumitrascu and Ciobanu, 2020 [32]	1. The noise exposure was above the legal limits of 87 dBA in Romania. 2. 13% of the cases had HAV exposure above 2.5 ms^−2^. 3. Dust exposure was within limits of 5 mgm^−3^. 4. Thermal stress was determined for workers to form wet bulb globe temperature. 5. Some 25.23% of the subjects had osteomusculoskeletal disorders, 0.93% with Raynaud’s syndrome and 3.74% had bilateral hearing loss.	HL

## Data Availability

Data sharing not applicable. No new data were created or analyzed in this study. Data sharing is not applicable to this article.
